# Recent artificial selection in U.S. Jersey cattle impacts autozygosity levels of specific genomic regions

**DOI:** 10.1186/s12864-015-1500-x

**Published:** 2015-04-16

**Authors:** Eui-Soo Kim, Tad S Sonstegard, Max F Rothschild

**Affiliations:** United States Department of Agriculture, Animal Genomics & Improvement Laboratory, Beltsville Agricultural Research Center, Agricultural Research Service, Beltsville, MD 20705 USA; Department of Animal Science, Iowa State University, Ames, IA 50011 USA

**Keywords:** SNP, Runs of homozygosity, Signatures of selection, Jersey cattle

## Abstract

**Background:**

Genome signatures of artificial selection in U.S. Jersey cattle were identified by examining changes in haplotype homozygosity for a resource population of animals born between 1953 and 2007. Genetic merit of this population changed dramatically during this period for a number of traits, especially milk yield. The intense selection underlying these changes was achieved through extensive use of artificial insemination (AI), which also increased consanguinity of the population to a few superior Jersey bulls. As a result, allele frequencies are shifted for many contemporary animals, and in numerous cases to a homozygous state for specific genomic regions. The goal of this study was to identify those selection signatures that occurred after extensive use of AI since the 1960, using analyses of shared haplotype segments or Runs of Homozygosity. When combined with animal birth year information, signatures of selection associated with economically important traits were identified and compared to results from an extended haplotype homozygosity analysis.

**Results:**

Overall, our results reveal that more recent selection increased autozygosity across the entire genome, but some specific regions increased more than others. A genome-wide scan identified more than 15 regions with a substantial change in autozygosity. Haplotypes found to be associated with increased milk, fat and protein yield in U.S. Jersey cattle also consistently increased in frequency.

**Conclusions:**

The analyses used in this study was able to detect directional selection over the last few decades when individual production records for Jersey animals were available.

**Electronic supplementary material:**

The online version of this article (doi:10.1186/s12864-015-1500-x) contains supplementary material, which is available to authorized users.

## Background

The genomes of modern cattle have been under constant selective pressure for a variety of traits valued by breeders, since domestication began nearly 14,000 years ago [1]. Some of the footprints of selection reflect the history of animal movements by migratory farmers out of the ancient centers of cattle domestication as well as selection within the past few centuries for breed type associated with milk or meat production [2]. Identifying the sequence variation underlying these footprints of selection may reveal genetic mechanisms with major effects on production differences within and between breeds. This information, in turn, may prove valuable for rapid improvement of less productive cattle populations. However, determining the most appropriate method to detect selective sweeps depends on a number of factors; some of which include origin of founder animals, genealogical history of the target population, and recency of selection events.

Many previous reports of selective sweeps in cattle were based on site frequency dependent analysis or examination of population sub-division, which is well-suited for demonstrating evidence of long-term natural selection [3]. Use of a population fixation index (*F*_*st*_) estimation has revealed genome-wide differences between bovine sub-species [4] and European dairy breeds developed from geographically distinct founder populations [5]. Likewise, examination of allele frequency differences between dairy and beef breeds was used to detect genomic regions under long-term selection for the different types of animal protein production [6]. However, neither of these methods seems well-suited to detect selection events that occurred more recently, after breed formation.

In human studies, extended haplotype analysis (EHH) was developed to detect more recent selection events [7], which is supported by detection of haplotypes at high frequency due to incomplete selective sweeps. A variation of the EHH approach, known as the integrated haplotype homozygosity (iHS), allows comparison between haplotypes that carried the ancestral and derived SNP alleles after summing extended haplotype homozygosity over all sites from a core SNP [8]. To extend these methods, Tang and colleagues [9] proposed an alternative application of EHH by contrasting the extended haplotype homozygosity profiles between divergent populations (R_sb_). This approach has been optimized for nearly fixed selective sweeps, whereas the iHS approach has higher power to detect partial sweeps [9]. In cattle, the R_sb_ method identified footprints of selection in the ancestral admixture between some African breeds by comparing EHH with the other distantly differentiated breeds derived from *Bos taurus* and *indicus* ancestry [10]. Despite the historical decay of haplotypes responsible for cattle breed formation, many selective sweeps were also revealed in Holsteins using EHH based methods [11,12]. A combination of all three methods (EHH, iHS, R_sb_) was also used to find selective sweeps between dairy and beef cattle breeds of both sub-species [13].

While some of the empirical differences between breeds could be explained by the selective sweeps detected in the aforementioned studies, consideration must be given to more recent systematic organized selection for improved production that has potentially changed the genome composition of a few intensely selected breeds. An example is the quantitative genetics-based selection practiced over the last five decades in popular dairy cattle breeds, where milk yields for contemporary cows is now about double that of predecessors from 50 years ago [14]. Selection for advantageous alleles with additive effects has improved production ability in an extraordinary and rapid way; especially with the co-emergence of reproductive technologies like artificial insemination (AI) where a few influential sires can generate tens of thousands of progeny. As breeders increase the frequency of beneficial alleles, inbreeding becomes an essential procedure [15]. Conversely, inbreeding of the locus under selection differs from the mean autozygosity of the whole genome, as is the case for quantitative trait loci (QTL) [16]. Thus, selection, inbreeding, and fixation of QTL appear to be dependent phenomena because artificial selection of superior animals can hardly be achieved without mating between related individuals carrying the superior founder alleles. However, identification of short-term artificial selection, especially within breed, has been a more elusive challenge; in part, due to a lack of representative DNA samples that capture breed diversity prior to the inception of AI and intense artificial selection for milk production.

One breed that has undergone this type of recent selection is Jersey cattle, which originated from the British Channel Island of Jersey prior to importation to the United States (US) and other countries during the mid 19^th^ century [17]. US Jersey cattle have been developed for high productivity, and identification of genomic regions under recent artificial selection would shed light on the genes affecting important economic traits. Although breeders have tried to minimize inbreeding by systematic mating plans in creating the American Jersey type, many of the important haplotypes affecting production have originated from only a few influential ancestors. This phenomena of a reduction in diversity persists when the offspring of influential sires tend to be selected by breeders with parallel objectives for genetic improvement, accelerating the similarity between descendants in a few generations [18]. Thus, it could be hypothesized that overall levels of genomic autozygosity have increased across US Jerseys.

In order to test this hypothesis and attempt to identify more recent selection signatures, we examined runs of homozygosity and haplotypes in Jersey cattle born after 1950s with phenotypic records, which enables monitoring of genomic autozygosity and haplotype frequency relative to changes in production. We then compared the results of ROH analysis with patterns of extended haplotype decay in an attempt to differentiate selection signatures resulting from artificial and natural selection. In addition genome-wide association scans between the most frequent haplotypes and traits were performed to ascertain evidence of artificial selection for increased milk, fat, and protein yield. Our results demonstrate how the combination of pedigree, traits, and haplotype analysis facilitates confirmation of evidence of recent selection signatures, providing insights into selection during the late 20th century aimed at improvement of dairy production traits.

## Methods

### Genotype and pedigree

Pedigree information and Illumina BovineSNP50 genotypes (Illumina, CA) for 1,602 US registered Jersey animals (1,219 sires and 383 dams) were obtained from USDA-ARS, Animal Improvement Programs Laboratory, and birthyear ranged from 1953 to 2007 (see Additional file 1A). No new animal samples were collected and no experimentation directly on animals was done to obtain these genotypes. These animals were members of the 80 half-sib families (size ≥3) from sires produced between the 1950s to 1990s. The recorded pedigrees for these 1,602 animals were used for estimating pedigree-based inbreeding coefficients, and encompassed 19,664 animals; some of which trace back to the 1940s. Most genotypes animals (see Additional file 1B) were produced during the 1990s (n = 814) or after 2000 (n = 469), while about 300 animals were born before the 1990. Most of the latter group are influential ancestors of all contemporary US Jerseys. In particular, five of the influential sires (genotyped) were common ancestors of ≥100 genotyped animals. A total of 37,154 markers located on autosomes were selected for the analysis based on a minor allele frequency (>0.01), call rate across animals (>0.8), and availability of UMD 3.1 genome coordinates.

### ROH and Locus autozygosity (F_L_)

An intact homozygous genomic region was defined by state of contiguous homozygous genotypes, which has been termed runs of homozygosity (ROH) [19,20]. The criteria for defining genomic regions as ROH has been a length of 50 or more consecutive homozygous SNPs (>1 Mb) considering the density of SNPs. ROH-based inbreeding coefficients were estimated to be highly correlated with a pedigree inbreeding coefficient (~0.7) using 30, 50, and 80 SNP windows [21]. Thus, we used a threshold of 50 consecutive homozygous genotypes to define ROH. Next, the intact homologous genomic region of an individual was assessed for each locus in the population. The locus homozygosity (autosomal only) was defined by the sum of locus autozygous status divided by total animals. Locus autozygosity (*F*_*L*_) is $\frac{{\displaystyle {\sum}_{l=1}^N}ro{h}_l}{N},$ where *N* is the total individuals (1,603), and *roh* is the autozygous status (0 or 1) of a SNP based on ROH of *l* individual.

### The change of locus autozygosity and haplotype frequency

We estimated ROH as a means to identify (across all haplotypes) each encompassing locus under artificial selection. In this analysis, directional selection for economically important dairy traits since 1960s was assumed. The change of locus homozygosity was modeled using logistic regression, and analysis was performed using locus homozygousity status as a response and animal birth year as a predictor. The logistic regression model was:

$$ ro{h}_l=\frac{1}{1+{e}^{-\left(a+\beta b\right)}}, $$

where, *roh*_*l*_ was the autozygous state of SNP locus *l*, *b* was animal birth year, α was the intercept of the equation, and *β* was the coefficient of the predictor.

Although ROH represented the sum of all haplotype-based homozygosity, it comprised a relatively small number of frequent haplotypes. After phasing using fastphase [22], a haplotype association test was performed across the genome. Association test haplotypes were determined using 50-SNP sliding window on the haplotype contributing to ROH. Using the logistic regression model, associations between animal birth year and the most frequent haplotype were evaluated in each sliding window. Statistical thresholds were determined empirically using permutation tests [23], and the experiment-wise critical values (top 1% and 5% *p* values) were obtained by 1,000 permutations.

### Signature of selection using extended haplotype homozygosity (iHS)

Evidence for positive selection was determined by calculating the value of the standardized integrated extended haplotype homozygosity (iHS) for each marker [8]. Ancestral alleles were derived from BovineSNP50 genotypes from a previous study [24]. The signed iHS was calculated as an unstandardized value, and then transformed to obtain genome-wide adjusted p-values, where the core SNPs (MAF ≥0.03) have a mean 0 and variance 1 [8]. Based on the ROH analysis, signatures of haplotype decay were investigated for 5 Mb of flanking genome from the core SNP. The absolute value of iHS ≥2 (p ≤0.01) indicates potential selection, and iHS ≥3 (p ≤0.001) is considered as significant evidence of recent selection, respectively. As a means to filter potential false positives, core SNP with iHS >3 and at least 10 other nearby (0.5 Mb) core SNPs with iHS >2 were considered further.

### Signature of selection using extended haplotype homozygosity (R_sb_)

Evidence for positive selection was also determined by calculating the value of EHH for each marker between two different animal groups. Group critieria was based on animal birth year, where the oldest 300 genotyped animals (18.8%) born before 1991 were defined as the ancestral group while an equal set of animal genotypes from the remaining 1,303 young animals represented a contemporary group (2001–2007). These two groups were contrasted to investigate changes of EHH between the 1980s and 2000s. The predicted transmitting abilities (PTAs) for milk yield, fat, and protein were 305.2 (±582.1), 16.6 (±21.7), and 11.3 (±15.6) in our contemporary animals, which was significantly higher than those for the ancestral group (milk yield = −941.9 (±697.6), fat = −33.9 (±28.1), protein = −33.5 (±21.2)).

R_sb_ analysis was done as described by Tang et al. [9], using a custom Perl script. Extended haplotype homozygosity (EHHS) was calculated separately with EHHS_A_ and EHHS_C_ for the ancestral and contemporary groups, respectively. Integrated EHHS (iES) between these two groups was compared by calculating accumulated EHHS size around the target SNP. The log ratio of the ancestral integrated EHH (iES_A_) and contemporary integrated EHH (iES_C_) was defined as iES_A_/iES_C_ = ln(R_sb_)’. The ln(R_sb_)’ value of each locus was standardized against the genome-wide data set as above for iHS.

### Associations between traits and haplotype

In order to investigate the impact of recent artificial selection, an association test was completed between haplotypes and dairy traits economic importance. The 19,664 animals of the extended pedigree was used to estimate milk yield, protein, and fat PTAs (USDA-ARS-AIPL) and assess the additive genetic effect of the haplotype. In this analysis, the most frequent haplotype was hypothesized to be under artificial selection if it carried the advantageous allele that contributed the most variation to the additive genetic effect of an economic trait. Haplotype associations were evaluated using generalized linear model, *y* = *μ* + *βG* + *e*, where *y* is PTA of an individual, *μ* is mean, and *β* is a vector of additive genetic effect. *G* is an indicator variable for the additive genetic effect of an individual, and *e* is a vector of individual error terms. To estimate the additive effect, the genetic effect of the other haplotypes (except the most frequent haplotype) was set to 0 [18]. Haplotype was defined using a 50-SNP sliding window, as above. In all analysis models, the genome-wide significance level (exceeding a significance threshold) was calculated using a permutation test with the experiment-wise error rate (1%) as a threshold [23]. For the all statistical analyses described above, R, Perl, and C were used.

## Results

### Genome-wide autozygosity

**Figure 1:**
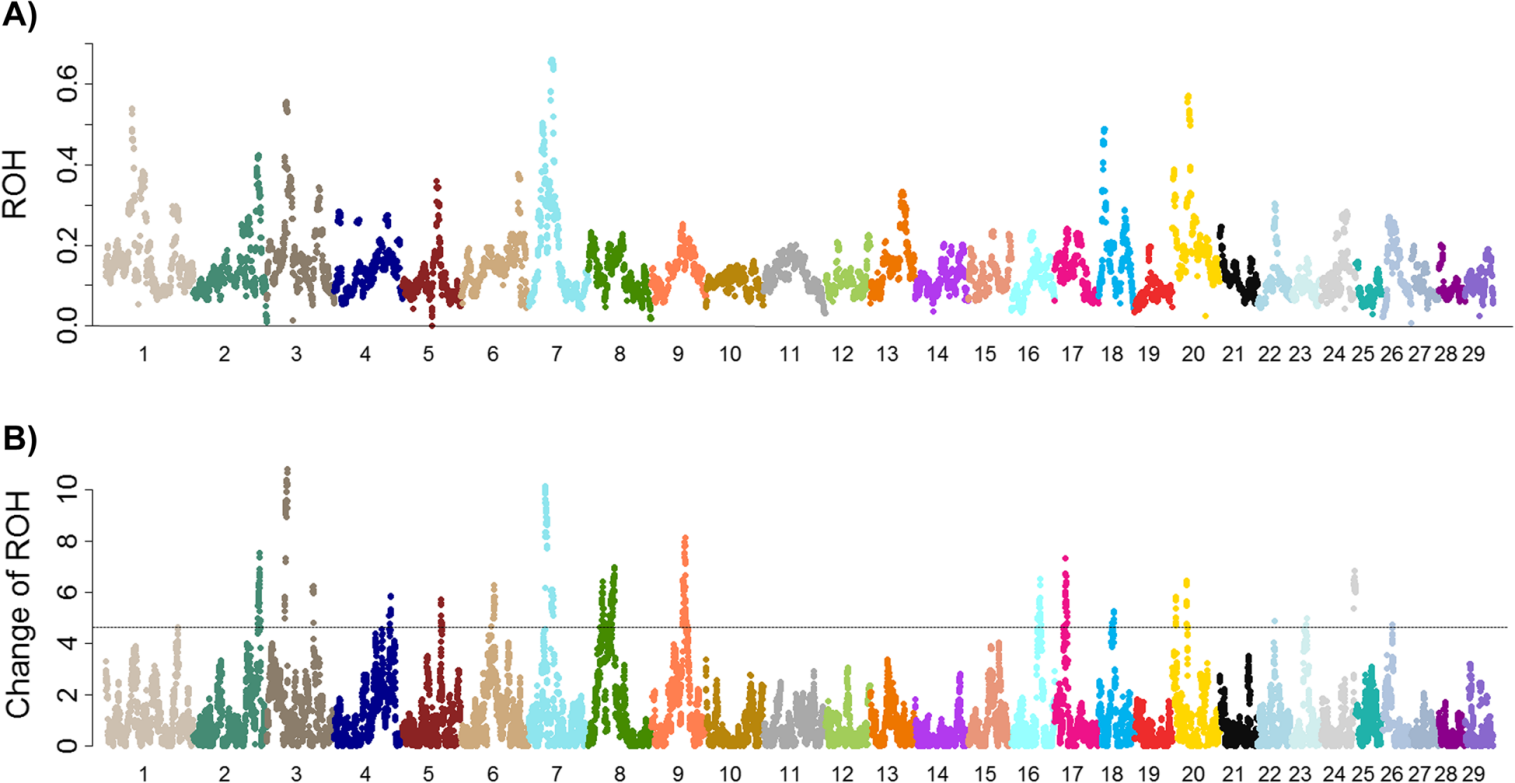
Manhattan plots of genome-wide *F*_*L*_ (**A**), and change of autozygosity Δ*F*_*L*_ (**B**). Genome-wide *F*_*L*_ (**A**) and significant levels of associations (−log10(p)) between ROH and birthyear (**B**) were plotted against SNP coordinates derived from the bovine reference genome assembly (UMD 3.1). For (**B**), Genome-wide suggestive (5%) and significant level (1%) are 4.4 and 7.3, respectively.

The genome-wide pattern of ROH in terms of autozygosity (*F*_*L*_) was determined to examine local autozygosity. Figure 1 displays the average autozygosity (*F*_*L*_) plotted against each SNP across generations. This analysis was applied to idenitify regional autozygosity with excessive ROH. Considering the whole genome, the mean and standard deviation of *F*_*L*_ were 0.137 and 0.073, respectively, which reveals that a large proportion of autozygosity varies across chromosomes and between individuals. For instance, the mean *F*_*L*_ of chromosome (Chr) 20 differed from that of Chr 19 significantly (0.21 vs 0.09, see Additional file 2). It was noted that the estimate of *F*_*L*_ was also variable within a chromosome (s.d. range 0.03-0.15), as some ROH was traceable to specific ancestral sire haplotypes that had significantly more recorded progeny in the US Jersey herdbook.

### Change of autozygosity

A genome-wide scan of change of autozygosity (*ΔF*_*L*_) detected more than 40 regions (0.01 adjusted *p*-value, size >0.1 Mb) potentially representing a considerable increase of autozygosity during the last five decades (Figure 1A, see Additional file 3). To support this supposition, the top 18 genomic regions of *ΔF*_*L*_ spanning at least 0.5 Mb were subjected to genome-wide association tests of autozygosity state against birthyear (Table 1). The results show autozygosity has changed significantly since the 1960s in several regions, including Chr 2, 3, 7, 8, 9, 16, 17, 20, and 24 (Figure 1B; see Additional file 4). However, not all regions with significant *F*_*L*_ or *∆F*_*L*_ showed a relatively strong correlation to birth year (Figure 1). For example, *F*_*L*_ was less than 0.2 in the region spanning 4 Mb between 29–33 Mb on Chr 7 during the 1970s, and increased to 0.6 in the individuals produced after 2004. This change resulted in moderate levels of *F*_*L*_ (0.40) in all animals across generations. The maximum value of *F*_*L*_ (0.66) located at 43 Mb was also subject to a significant change of autozygosity (*∆F*_*L*_). However, *∆F*_*L*_ at the loci with maximum *F*_*L*_ was less significant than the signals at 29–33 Mb due to a high degree of existing autozygosity (*F*_*L*_ = 0.45) during the 1970s-80s. As expected, we obtained a positive coefficient corresponding to the increased autozygosity in most genomic regions that are significantly associated with birth year (adjusted p ≤ 0.01, Table 1). The pattern of *F*_*L*_ across generations appeared weakly related to *∆F*_*L*_. Conversely, several regions with high levels of ROH have been maintained without a large amount of change for decades (Figure 1). These regions reflect regions under producer-based artificial selection during the mid-20^th^ century or inbreeding status maintained following breed formation in the 19^th^ century.

**Table 1:** **Change of autozygosity (** ***ΔF***_***L***_ **)**

**BTA**	**Region** ^**1**^	**Maximum association (−** ***log***_***10***_ ***p*** **)**	**Direction of change (slope)**	**Maximum** ***F***_***L***_ **(Mb)** ^**2**^	**|iHS|** ^**3**^	**R**_**sb**_^**3**^
2	124.89-129.81	7.54	0.044	**0.42**	3.54	7.59
3	38.42-44.08	10.80	0.049	**0.56**	3.49	5.33
4	100.40-101.94	5.84	0.049	0.18	-	4.09
5	85.41-86.46	5.72	0.057	0.15	-	-
6	57.15-59.29	6.28	0.055	0.16	-	-
7	29.59-33.53	10.14	0.054	**0.40**	3.70	-
	40.27-45.76	6.09	0.035	**0.66**	-	-
8	26.73-31.06	6.42	0.061	0.13	-	-
	38.84-50.22	6.98	0.057	0.18	-	3.27
9	60.46-72.29	8.13	0.065	0.25	-	5.64
16	54.25-61.14	6.28	0.056	0.18	-	3.89
17	14.84-19.94	7.35	0.055	0.24	-	-
18	23.17-26.44	5.28	0.043	0.20	-	4.47
20	5.06-6.14	5.76	0.043	0.24	-	5.44
	20.99-22.88	6.45	−0.037	0.31	-	5.44
23	25.92-27.98	4.64	0.046	0.15	-	-
24	57.92-61.46	6.86	0.064	0.13	-	4.35
26	19.03-19.81	4.73	0.047	0.15	3.37	4.91

^1^Candidate regions are selected based on genome-wide 5% levels (genomic region > 0.5 Mb).

^2^Position of maximum locus homozygosity located in the candidate region defined by the change of homozygosity with bold highlights for regions >0.40.

^3^Maximum standardized R_sb_ value in the region (only iHS > 3, |R_sb_| > 3).

### Extended haplotype homozygosity

**Figure 2:**
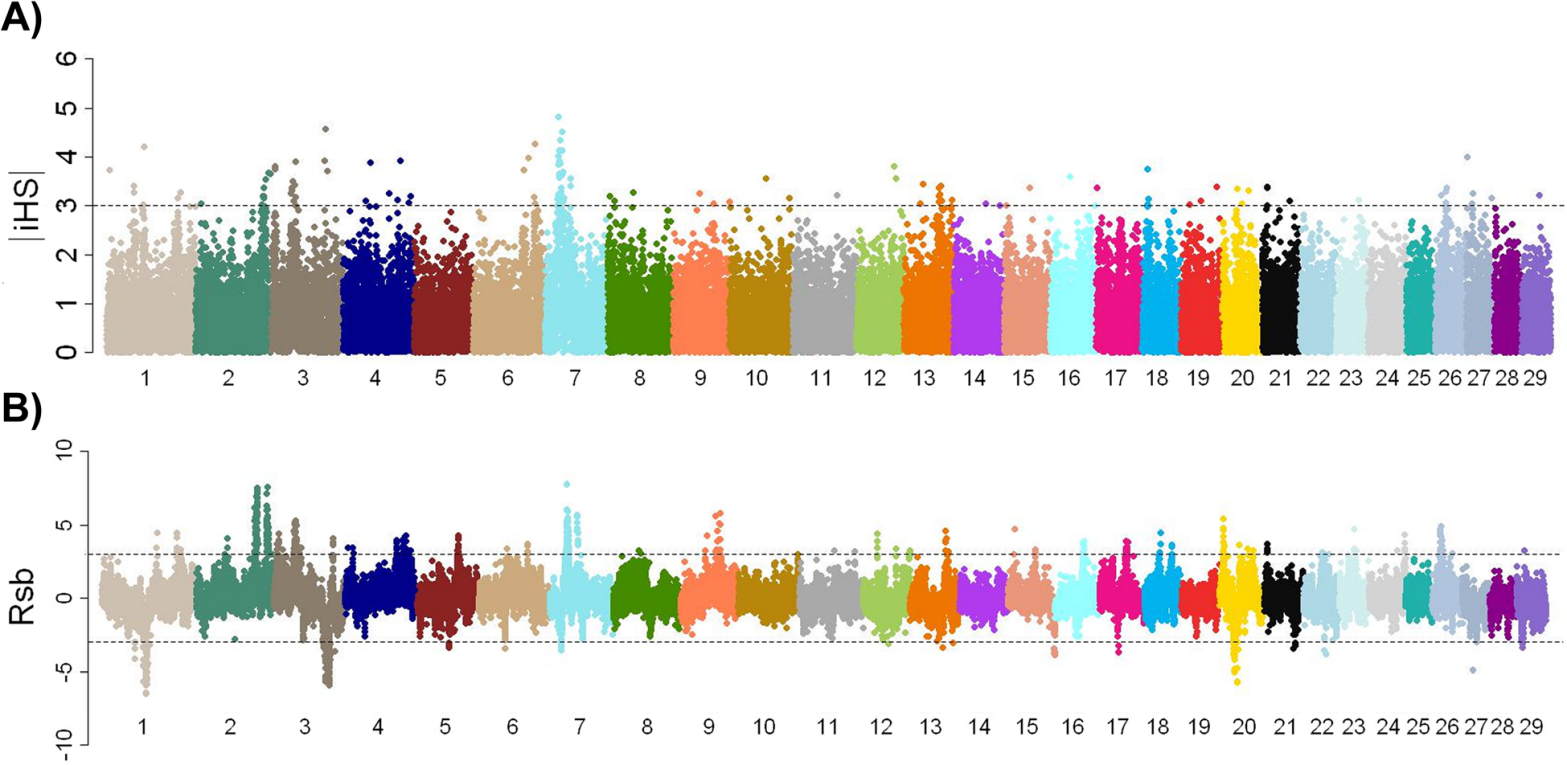
Genome-wide plots of |iHS| (**A**) and R_sb_ (**B**). The levels of |iHS| (**A**) and R_sb_ (**B**) were plotted based on SNP coordinates derived from the bovine reference genome assembly (UMD 3.1). Dotted line represents threshold level of iHS = 3 in (**A**) and R_sb_ = ±3 in (**B**).

A total of 1,211 autosomal loci were potentially subjected to selection (iHS ≥2) (Figure 2A; see Additional file 5). The maximum value of iHS was 4.83 flanked by >80 loci located between 21.9 and 31.2 Mb on Chr 7 (Table 2). The other candidate regions on Chr 1, 2, 3, and 18 were consistent with more recent “selection-based” regions of ROH. Of particular note, there were many significant iHS in a broad region between 4.8-8.2 Mb on Chr 3, which were discovered only by iHS. In contrast, specific regions on Chr 6, 13, and 26 overlapped with consistently higher levels of ROH probably derived during breed formation or breed improvement prior to the late 20^th^ century.

**Table 2:** **Summary of the standardized iHS value** ^*^

**BTA**	**Region (Mb)**	**Size (Mb)**	**|iHS| >2**	**Maximum |iHS|**	**Position (Mb)**
1	127.91-133.08	4.75	24	3.16	132.59
2	124.12-129.87	6.13	40	3.54	128.93
3	4.23-7.46	3.31	24	3.80	6.63
3	41.22-42.56	1.35	12	3.49	42.62
6	103.05-106.09	4.85	18	3.19	103.45
7	23.57-32.73	9.28	84	4.83	25.17
13	62.77-67.46	4.50	17	3.38	63.22
18	9.00-10.37	1.32	10	3.02	9.29
26	17.25-19.61	3.13	19	3.30	19.30

^*^Regions encompassing at least one |iHS| >3 and 10 flanking loci with |iHS| >2 are summarized.

After lowering the MAF threshold (0.02) to discover nearly fixed alleles, the standardized value of R_sb_ was calculated using the maximum 5 Mb flanking genome bracket size. Comparisons of the EHH between founder and contemporary groups revealed 2,077 loci (|R_sb_| >2) under selection across the genome (Figure 2B; see Additional file 6). A total of 1,387 (67%) had positive values of R_sb_ (R_sb_ ≥2) that represented the consequence of potential positive selection. The strongest evidence of positive selection was located at 31 Mb on Chr 7, agreeing with *∆F*_*L*_ and iHS analyses (see Additional file 7). Candidate regions grouped to have at least 20 SNPs with |R_sb_| ≥2 in the 200 kb region that includes one or more SNP with |R_sb_| ≥3 could differentiate between more recent and breed formation selection. Although a few candidate regions were unique, several large R_sb_ analysis signals (R_sb_ ≥3) overlapped with regions corresponding to *∆F*_*L*_ (Table 1; see Additional file 7). Moreover, we observed several regions with autozygosity in decline on Chr 1, 3, 20, and 29 (see Additional file 8), while most other regions only showed indications of selection that increased the autozygosity.

### Haplotype-trait association

**Figure 3:**
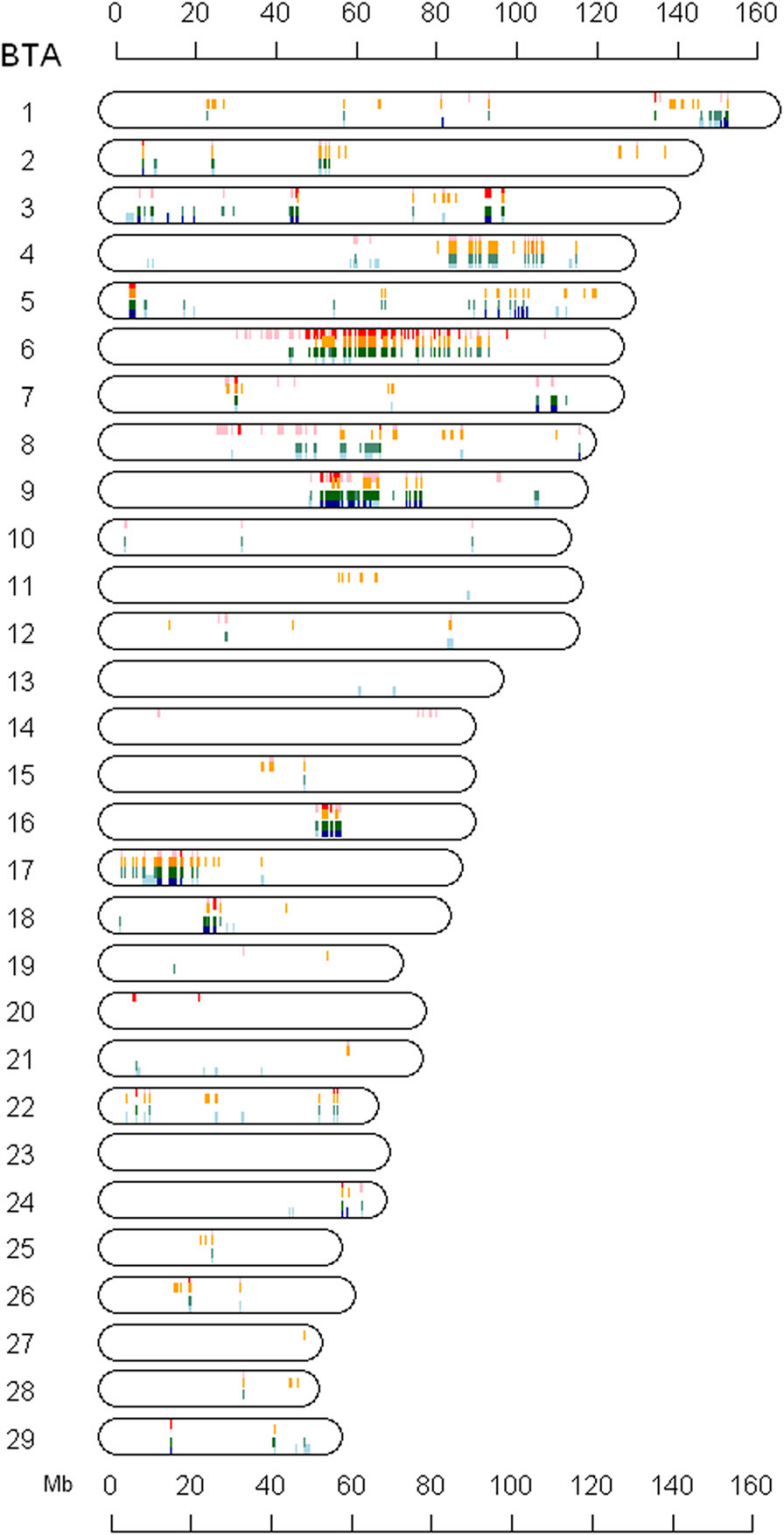
Genome diagram of associations between the most frequent haplotype and birth year, milk, fat and protein yield. Graphical representation of the bovine autosomes to demonstrate co-localized positions of haplotypes under selection and/or associated with milk production traits. Colored regions represent significant change of autozygosity (red/pink), and haplotype associations with fat (orange/light orange), protein (green/light green), and milk yield (blue/light blue). Color of the darker shade indicates candidate regions with significant association (genome-wide 1%), while the lighter shades represent suggestive associations (genome-wide 5%).

The haplotype-trait associations correlated well with the regions associated with milk yield, fat, and protein, describing the additive genetic effect of only the most frequent haplotypes (Figure 3; see Additional file 8). Altogether, *∆F*_*L*_ was largely concordant with the results from the associations between the most frequent haplotype and traits (Figure 3). Furthermore a substantial *∆F*_*L*_ was found that reflects the change of the most common haplotype relative to animal birth year. As a consequence, the most frequent haplotypes that increased persistently across the genome during the last five decades were bound to production of milk, fat, and protein.

In order to examine whether high levels of ROH affect phenotype, haplotype-trait associations were compared in the regions with *F*_*L*_ ≥0.3 and a significant *∆F*_*L*_ (Table 3). Interestingly, the region showing constantly high levels of *F*_*L*_ was not bound to milk yield. An abundant ROH generated in these cases might be related to advantageous alleles influencing other economic traits of importance before the more recent emphasis on the value of milk yield. We also observed loci under positive selection (iHS > 3) overlapping regions of *F*_*L*_ ≥ 0.3. These included regions on Chr 1, 3, 6, and 13 (Table 1). Nevertheless, autozygosity has not changed substantially with time in those regions, resulting in no association between the most frequent haplotype and traits.

**Table 3:** **Comparisons of |iHS|, R**_**sb**_ ***,*** **and** ***F***_***L***_

**BTA**	**Region with high** ***F***_***L***_ **(Mb)** ^**1**^	**Maximum** ***F***_***L***_	**Maximum |iHS|** ^**2**^	**Maximum |R**_**sb**_ **|** ^**3**^	**Major allele frequency** ^**4**^
1	44.47-54.63	0.31	2.69	-	0.73
	65.03-74.10	0.38	**3.03**	-	0.78
	130.90-131.75	0.30	**3.16**	2.08	0.79
2	122.33-129.80	0.43	2.88	-	0.65
3	38.42-53.96	0.55	**3.49**	**5.33**	0.88
	95.05-98.50	0.32	2.19	**4.09**	0.95
5	76.03-78.57	0.35	**-**	2.34	-
6	102.69-105.15	0.38	**3.19**	-	0.75
7	23.47-33.53	0.50	2.65	2.15	0.89
	35.57-48.41	0.66	2.81	**5.70**	0.70
13	60.06-66.55	0.33	**3.38**	**4.91**	0.59
18	7.39-10.33	0.49	**3.02**	-	0.72
20	1.43-5.03	0.39	2.65	**4.24**	0.93
	21.17-29.90	0.57	**3.36**	-	0.88

^1^Genomic region (>1 Mb) with high levels of ROH (*F*_*L*_ > 0.3)

^2^The maximum (iHS > 2) in the interval is shown. iHS > 3 is highlighted in bold.

^3^The maximum (|R_sb_| > 2) in the interval is shown. |R_sb_| > 3 is highlighted in bold.

^4^Major allele frequency of the loci with maximum iHS.

### Genes under selection

When considering the function of genes located near the candidate regions, positive selection of genes on Chr 7 (*CSNK1G3*, 27.8 Mb), and 18 (*CSNK2A2*, 26.0 Mb) are of particular interest since casein genes are known to be associated with economically important traits, particularly protein content in milk. Casein kinases (*CSNK1G3, CSNK2A2*) play a prominent role for phosphorylation of milk proteins occurring before micelle formation and milk secretion [25], and Kappa-casein (*CSN3*), which is located near the candidate region on Chr 6 (<1 Mb), is known as a crucial protein in cheese production. Signals from all analyses (iHS, R_sb_ and ROH) suggested a consensus footprint of selection on Chr 2 encompassing 125–130 Mb, which overlaps with a Holstein signature of selection in the vicinity of *E2F2* [26]. The *E2F2* gene is a member of *E2F* family of transcription factors that mediate mammary gland development in the mouse [27].

Resistance to infectious disease has not been a principal objective of genetic improvement in dairy cattle; however, selection for production traits can indirectly affect variation influencing disease resistance. The region encompassing *TLR1, TLR6*, and *TLR10* (60.5-61.0 Mb) on Chr 6, a region likely under recent selection, was found to be associated with susceptibility to clinical mastitis and somatic cell count in dairy cattle [28]. Toll-like receptors (*TLR*) are known to be involved in the innate immune system [29] participating in primary defense against bacterial infections that cause mastitis. The list of genes that appeared to be under recent selection and its functions are summarized in Additional file 9. According to biological databases including KEGG (www.kanehisa.jp/) and Wikipathways (www.wikipathways.org/index.php/WikiPathways), genes that were found in several candidate regions participate in starch and sucrose metabolism (*SMARCA2, UGDH, ENTPD7, HK3, DDX41, AGL*), B cell receptor signaling pathway (*MAP3K7, RPS6, RIK3AP1, CHUK, DOK3, NCK1, RPS6KA1*) and several pathways, particularly immune responses, biosynthesis and metabolism (see Additional file 9).

## Discussion and conclusions

In order to define genomic regions under recent selection, we first genome-wide ROH patterns in all animals as an initial analysis. In the late 20^th^ century, US Jersey genetic improvement for dairy traits relied on a few superior bulls. In practice, those alleles can be traced to origin by comprehensive genotyping of the pedigree, but expending those types of resources is not possible considering the cost and non-availability of DNA representing maternal lines. Furthermore, current algorithms for computing identical by descent (IBD) have been developed for only simple pedigrees, so determining IBD within complex pedigrees results low accuracies and high computational load. Although haplotypes are likely to break down in a few generations, some are maintained intact for generations because of limited crossover events. According to a previous human study, crossovers occur once on average within a 1-kb interval for every 90,000 gametes [30]. The length of autozygous segments is expected to be 100/(2 X [generations to a common ancestor]) cM [31], implying the existence of relatively large amounts of autozygosity in Jerseys. Moreover, number and length of ROH appear to be determined by the recent crossovers events [19]. Therefore, determining ROH in large complex pedigree is a feasible approach to examine autozygosity, which has emerged by selection and inbreeding.

The benefits of extended haplotype-based analyses include the ability to screen animal genomes regardless of birth year. In human studies, EHH is useful for detecting recent selection within the last 30,000 years [32], and the value of iHS is sensitive to the length of the ancestral haplotypes at each position [33]. Although a long extended haplotype could have been generated more than 100 years ago in Jerseys, iHS detects selected regions with no considerable allele frequency changes during the last 50 years, especially if it was nearly fixed during the early 20^th^ century. The regions surrounding loci with iHS >3 were rarely correlated to milk yield, implying that EHH was created prior to the systematic quantitative genetics-based breeding programs launched in the 1960s. While genotype frequencies change due to inbreeding, allele frequencies do not change apparently without selection [34]. Since inbreeding affects all loci equally and genetic drift changes frequency of loci randomly, inbreeding may not induce linkage disequilibrium between neighboring loci, whereas selection will drive linked alleles to high frequency [6]. Even if there is no strong selection of a specific allele, linked genomic regions of an individual could correspond to non-uniform inbreeding [35]. Thus, we also examined the change of haplotype frequency during the last five decades to better interpret our EHH results. The spectrum of results from R_sb_ deviate from that of iHS, with an excess of nearly fixed alleles, whereas iHS tends to have an excess of alleles with frequency below 0.9 [9].

Despite successful application of EHH-based methods, it is noteworthy that the core allele frequency or length of an extended haplotype embracing the selected allele provides limited context for receny of selection. During the last few centuries, artificial selection and AI have decreased the effective population size of popular dairy breeds rapidly with estimates of linkage disequilibrium [36], reflecting increased autozygosity. Inbreeding can generate correlations between loci throughout the genome [37], but it cannot create clusters of high frequency derived alleles without the occurrence of other genetic events such as selection or migration [6]. In small populations, fluctuations in allele frequency appear to occur from one generation to the next by chance [38], and allele frequencies also change whenever individuals have different numbers of offspring. An extreme change of allele frequency occurs due to population bottlenecks that dominate the process of random drift in the long term [39]. For the purpose of breeding, about 10 influential sires from the 1960s transmitted their alleles to more than 1,000 descendants in our pedigree of 19,664 animals, and apparent local and overall autozygosity are now observed across the genome. Individuals with advantageous or adaptive alleles tend to be more successful than the others with respect to increased reproduction in a contemporary group [40]. With the assistance of AI, superior sires are now able to migrate globally by passing on their alleles to succeeding generations more than natural service sires. By the early 20^th^ century, animal breeders might have been indirectly selecting alleles that were associated with improved milk production. Many of regions harboring high ROH are in founding animals, and have been maintained for decades. It is therefore not surprising that milk yield is weakly correlated to the regions with consistently high levels of ROH (*F*_*L*_ >0.3).

When comparing dairy cattle breeds, Jersey and Holsteins seem to originate from different recent ancestry according to genetic distances [41]. Specifically, the primary objective of selection has been high percentage of fat and protein in Jersey. Nevertheless, a selection signature on Chr 2 at ~128 Mb appears to overlap to a region with a high degree of ROH in US Holsteins [18], as well as the signature of selection in German Holsteins [26]. This genomic region is more likely to have been selected before the 1960s in US Holsteins, whereas the corresponding region has been under the influence of recent selection in Jerseys. Interestingly, our results, which suggest recent selection of specific alleles for casein genes such as *CSNK1G3* and *CSNK2A2*, are relatively in agreement with economic trends in US dairy component traits. For example, milk pricing was based on milk volume and fat content until 1980s, when economic value shifted from fat to protein. Our results appear to capture this shift in emphasis from fat to protein. We expect as the value of milk components continues to change so will autozygosity in specific genomic regions underlying these traits. Of note, the value of fat in selection was recently changed again by the Amercian Jersey Cattle Association (USDA-AIPL, https://www.cdcb.us/reference/nmcalc.htm#Yield).

While the history of cattle domestication has extended over thousands of years, the extraordinary improvement of milk yield has been achieved in dairy cattle during the 20^th^ century. Even though recent selection contributed strongly to improvement in production, many beneficial alleles appear to originate from quite old ancestors, as illustrated by the K232A mutations in *DGAT1* underlying a QTL with significant effects on dairy traits and yet still segregating in dairy cattle breeds [5]. To identify selection signatures of old favorable alleles, the population maintained on the Jersey Island may unravel clues to elucidate genomic regions involved in breed formation [42].

We analyzed selection and associations separately to assess the effects of selection on quantitative traits. The combination of genome-wide associations and signatures of selection based on the same set of SNPs help facilitate our ability to unravel loci influencing complex traits [43,44]. A previous study [45], which is concordant with our results, reported that the changes in haplotypes frequencies in sires accurately estimated genetic trends in the commercial cow population and could be applied to detect signatures of recent selection in Israeli Holsteins. Considering the candidate regions under selection and at the same time accounting for increased milk yield have provided the genomic status of the contemporary population of US Jerseys, suggesting the fundamental genotypic parameters available for the future breeding plans balancing productivity and diversity. Conclusively, we have suggested approaches to distinguish the recent signature of selection from an old evidence of selection. The results in our study also give an insight to correlations between haplotype, autozygosity, target traits, and objectives of selection in dairy breeding.

### Availability of supporting data

The genotype and phenotype data and animal identification information analyzed in this manuscript were provided to us for research purposes by partners in the North Amercian dairy industry. This group and all its data are now under the supervision of the Council for Dairy Cattle Breeding. Supporting data are available by request to the Council on Dairy Cattle Breeding, 6486 E Main Street, Reynoldsburg, OH 43068; Ph: 614 861 3636 x4469, Fax:614 861 8040.

## Additional files

Additional file 1:
**Bar graph plots of Jersey animals by birth year.** This figure is a plot of the number of Jersey animals (y-axis) in the pedigree (A) or with genotypes (B) used in this study binned by birth year (x-axis). Additional file 2:
**Mean and maximal locus autozygosity (** ***F***_***L***_ **) values for bovine autosomes.** This table lists the mean and maximal values of (*F*_*L*_) obtained for each autosome, and the genome coordinate for each maximal value is also shown. Additional file 3:
**Chromosomal autozygosity (** ***F***_***L***_ **) plots.** This figure shows chromosomal plots of the magnitude (y-axis) of *F*_*L*_ for each bovine autosome based on SNP coordinate (x-axis). The grey shaded bars indicates (*F*_*L*_) under 0.2, while the red bars show where *F*_*L*_ exceeds 0.3. Additional file 4:
**Chromosomal plots of change in autozygosity (**Δ***F***_***L***_ **).** This figure shows chromosomal plots of the values (y-axis) of Δ*F*_*L*_ (−log10p) based on associations of autozygosity and birth year for each loci relative to SNP genome coordinate (x-axis). The grey shaded bars indicate Δ*F*_*L*_ values not exceeding the genome-wide significance level (*p* = 0.01), while those plotted in red reach this threshold. Additional file 5:
**Chromosomal plots of |iHS| values.** This figure shows chromosomal plots of the absolute value of standardized iHS (y-axis) for each loci relative to SNP genome coordinate (x-axis). Those |iHS| exceeding 3.0 are highlighted in red. Additional file 6:
**Chromosomal plots of R**_**sb**_ **values.** This figure shows chromosomal plots of the values of standardized R_sb_ (y-axis) for each loci relative to SNP genome coordinate (x-axis). Those R_sb_ exceeding an absolute value of 3.0 are highlighted in red. Additional file 7:
**Genome diagram for comparison of significant iHS, R**_**sb**_ **and change of autozygosity (**Δ ***F***_***L***_ **) values.** Bovine autosomes are shaded to represent approximate genome locations where significant regions were detected for changes in autozygosity (adjusted p = 0.01, dark red color; adjusted p = 0.05, light red color), iHS (dark blue >3.0, light blue >2.5), and R_sb_ (dark orange >3.0, light orange > 2.0). Additional file 8:
**Genomic regions with significant R**_**sb**_ **values.** This table summarizes all genomic regions with |R_sb_| >3. Additional file 9:
**List of gene positions and functions within candidate regions under selection.** The gene positions (Table S1) located in genomic regions determined to be under recent selection regions are summarized. The gene function information (Table S2) was also summarized based information obtained from KEGG and Wikipathways database.
