# Supplementary figures and images for: Recent artificial selection in U.S. Jersey cattle impacts autozygosity levels of specific genomic regions
Source: BMC Genomics. 2015 Apr 16;16(1):302. doi: 10.1186/s12864-015-1500-x (PMC4409734; doi:10.1186/s12864-015-1500-x)

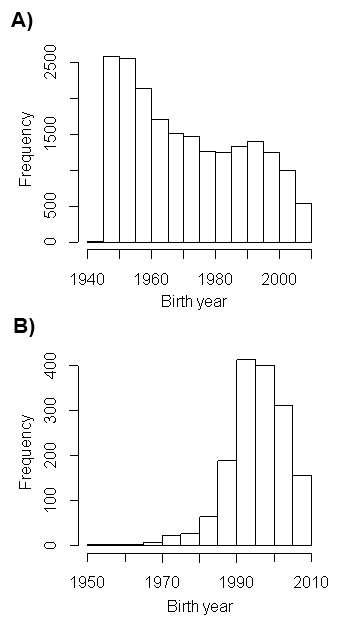

Supplement: Additional file 1: — Bar graph plots of Jersey animals by birth year. This figure is a plot of the number of Jersey animals (y-axis) in the pedigree (A) or with genotypes (B) used in this study binned by birth year (x-axis). [file 12864_2015_1500_MOESM1_ESM.tiff]

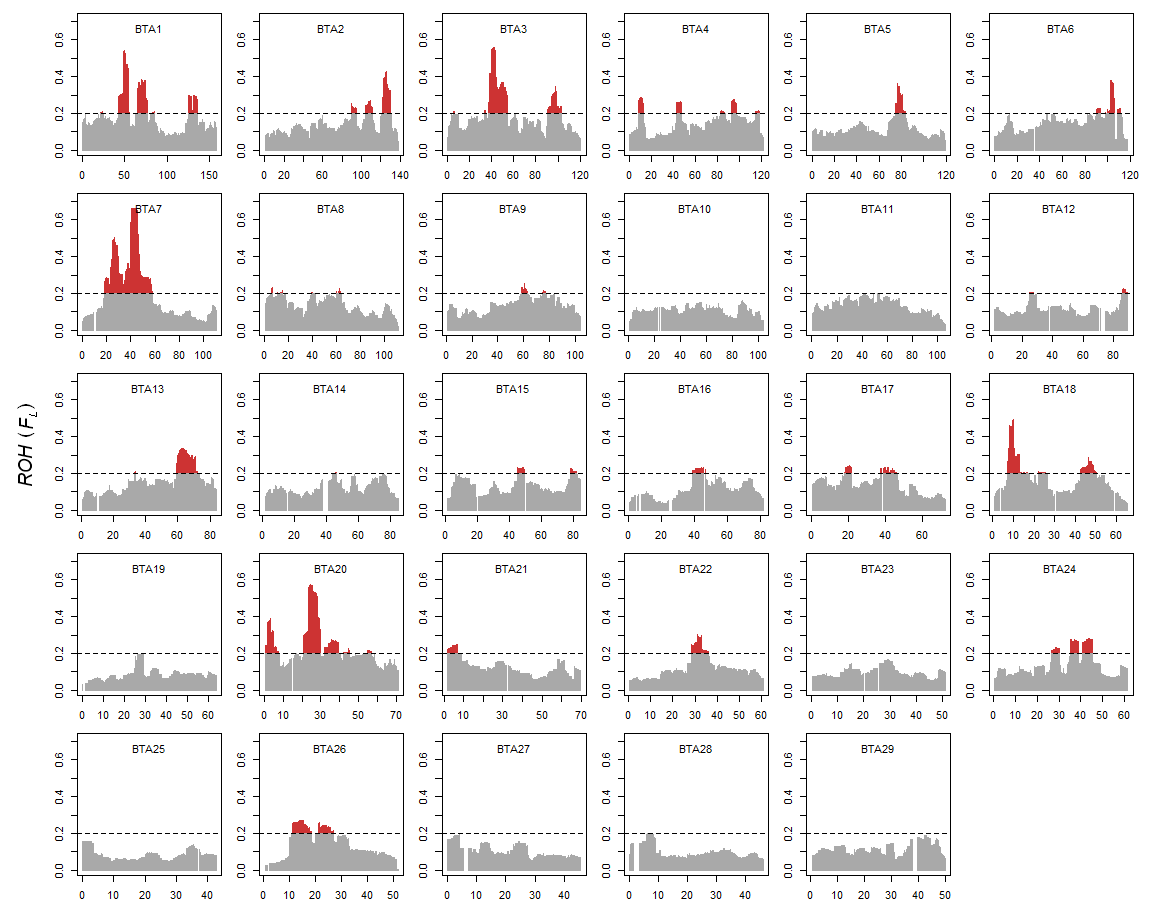

Supplement: Additional file 3: — Chromosomal autozygosity ( F L ) plots. This figure shows chromosomal plots of the magnitude (y-axis) of F L for each bovine autosome based on SNP coordinate (x-axis). The grey shaded bars indicates (F L) under 0.2, while the red bars show where F L exceeds 0.3. [file 12864_2015_1500_MOESM3_ESM.tiff]

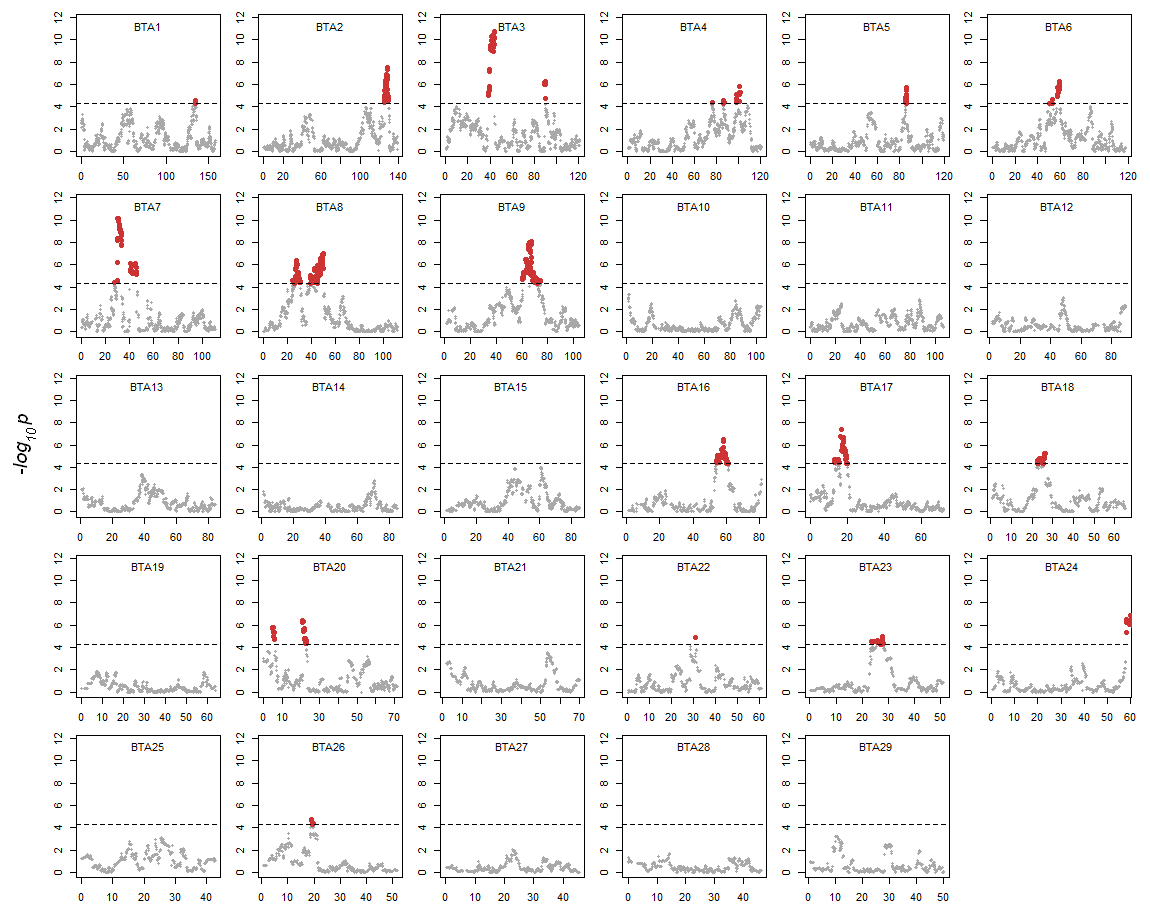

Supplement: Additional file 4: — Chromosomal plots of change in autozygosity (ΔF L ). This figure shows chromosomal plots of the values (y-axis) of ΔF L (−log10p) based on associations of autozygosity and birth year for each loci relative to SNP genome coordinate (x-axis). The grey shaded bars indicate ΔF L values not exceeding the genome-wide significance level (p = 0.01), while those plotted in red reach this threshold. [file 12864_2015_1500_MOESM4_ESM.tiff]

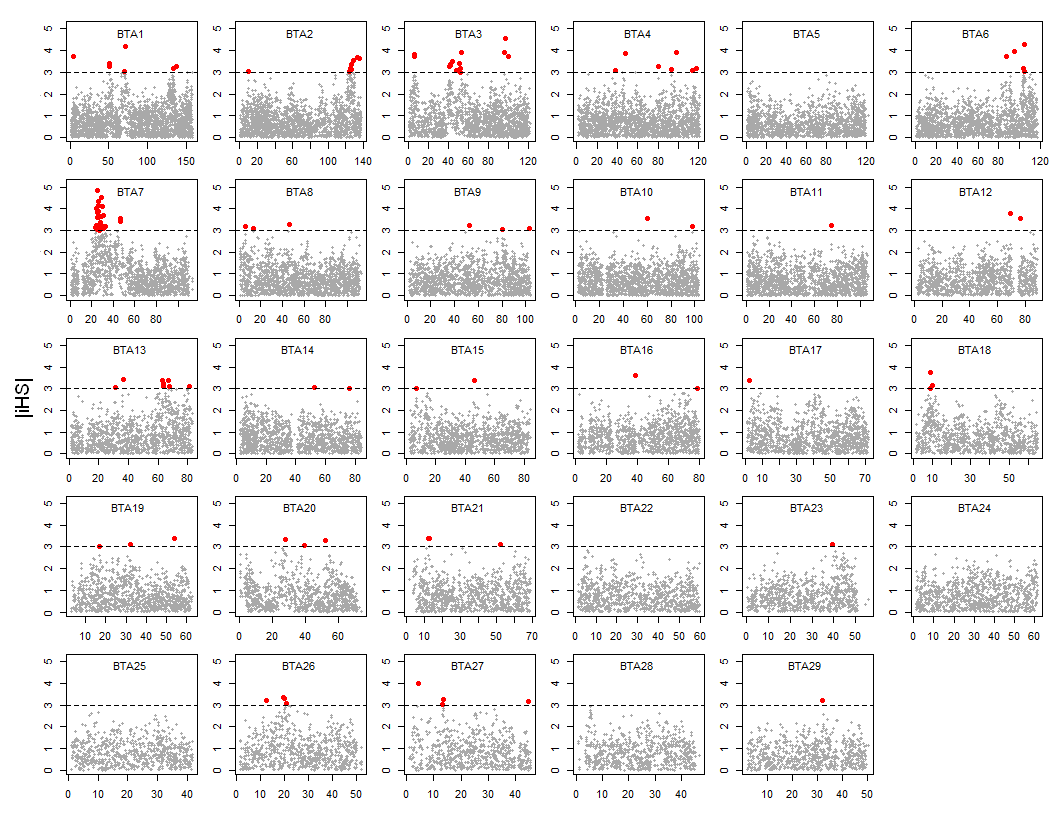

Supplement: Additional file 5: — Chromosomal plots of |iHS| values. This figure shows chromosomal plots of the absolute value of standardized iHS (y-axis) for each loci relative to SNP genome coordinate (x-axis). Those |iHS| exceeding 3.0 are highlighted in red. [file 12864_2015_1500_MOESM5_ESM.tiff]

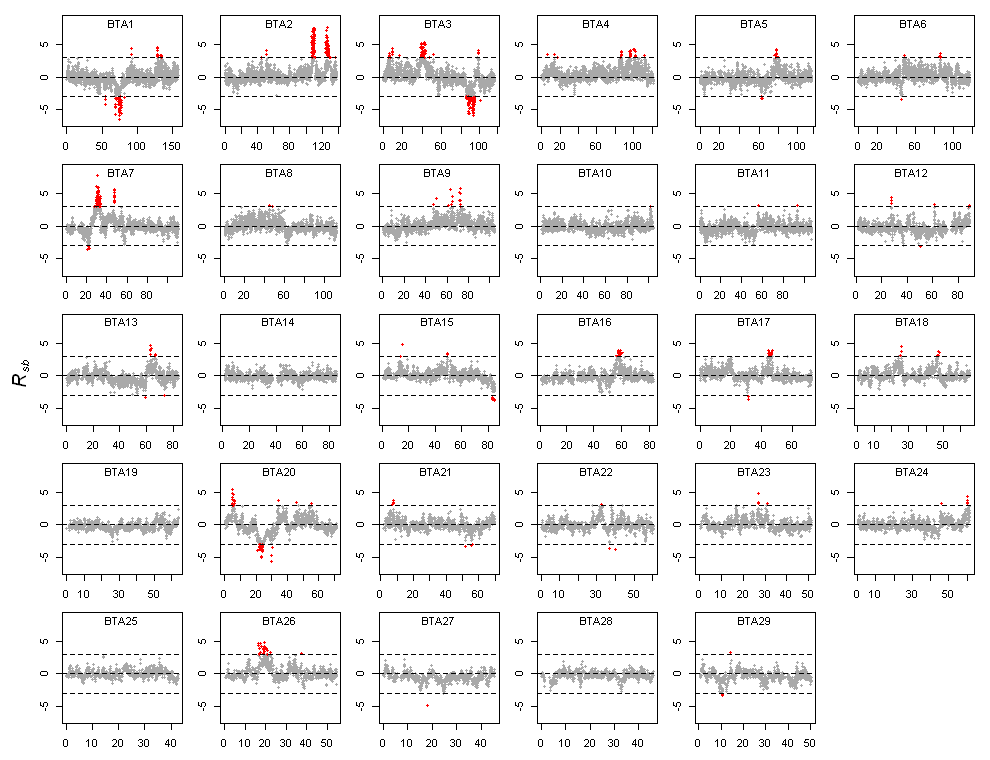

Supplement: Additional file 6: — Chromosomal plots of R sb values. This figure shows chromosomal plots of the values of standardized Rsb (y-axis) for each loci relative to SNP genome coordinate (x-axis). Those Rsb exceeding an absolute value of 3.0 are highlighted in red. [file 12864_2015_1500_MOESM6_ESM.tiff]

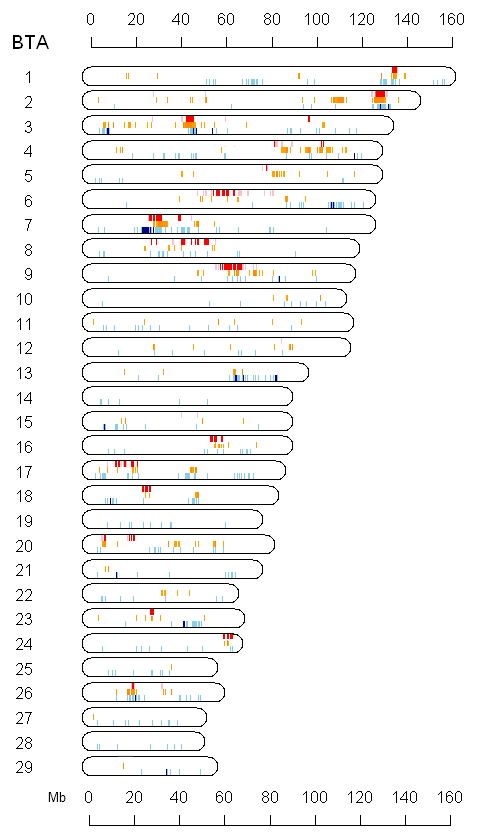

Supplement: Additional file 7: — Genome diagram for comparison of significant iHS, R sb and change of autozygosity (Δ F L ) values. Bovine autosomes are shaded to represent approximate genome locations where significant regions were detected for changes in autozygosity (adjusted p = 0.01, dark red color; adjusted p = 0.05, light red color), iHS (dark blue >3.0, light blue >2.5), and Rsb (dark orange >3.0, light orange > 2.0). [file 12864_2015_1500_MOESM7_ESM.tiff]
