# Supplementary material for: Recent artificial selection in U.S. Jersey cattle impacts autozygosity levels of specific genomic regions
Source: BMC Genomics. 2015 Apr 16;16(1):302. doi: 10.1186/s12864-015-1500-x (PMC4409734; doi:10.1186/s12864-015-1500-x)
Supplement: Additional file 2: — Mean and maximal locus autozygosity ( F L ) values for bovine autosomes. This table lists the mean and maximal values of (F L) obtained for each autosome, and the genome coordinate for each maximal value is also shown. [file 12864_2015_1500_MOESM2_ESM.docx]

**Additional file 2. Mean and maximal locus autozygosity (*F_L_*) values for bovine autosomes.**

| **BTA** | **Mean *F_L_*** | **Standard deviation** | **Maximum *F_L_*** | **Position (Mb)^*^** |
| --- | --- | --- | --- | --- |
| 1 | 0.17 | 0.09 | 0.54 | 49.75 |
| 2 | 0.14 | 0.07 | 0.43 | 125.75 |
| 3 | 0.18 | 0.10 | 0.56 | 41.82 |
| 4 | 0.14 | 0.06 | 0.28 | 102.41 |
| 5 | 0.11 | 0.06 | 0.36 | 76.09 |
| 6 | 0.15 | 0.06 | 0.38 | 102.69 |
| 7 | 0.19 | 0.15 | 0.66 | 40.83 |
| 8 | 0.13 | 0.05 | 0.23 | 6.15 |
|  | 0.13 | 0.04 | 0.25 | 60.70 |
| 10 | 0.11 | 0.02 | 0.16 | 88.09 |
| 11 | 0.13 | 0.04 | 0.20 | 53.10 |
| 12 | 0.12 | 0.04 | 0.23 | 86.40 |
| 13 | 0.16 | 0.07 | 0.33 | 62.63 |
| 14 | 0.12 | 0.04 | 0.20 | 45.81 |
| 15 | 0.13 | 0.04 | 0.24 | 49.06 |
| 16 | 0.11 | 0.05 | 0.23 | 45.40 |
| 17 | 0.14 | 0.05 | 0.24 | 20.12 |
| 18 | 0.17 | 0.09 | 0.49 | 9.92 |
| 19 | 0.09 | 0.03 | 0.20 | 26.64 |
| 20 | 0.21 | 0.10 | 0.57 | 24.65 |
| 21 | 0.12 | 0.04 | 0.25 | 6.56 |
| 22 | 0.11 | 0.05 | 0.30 | 31.08 |
| 23 | 0.10 | 0.03 | 0.17 | 29.23 |
| 24 | 0.14 | 0.07 | 0.28 | 43.90 |
| 25 | 0.10 | 0.03 | 0.16 | 2.27 |
| 26 | 0.14 | 0.07 | 0.27 | 14.24 |
| 27 | 0.11 | 0.03 | 0.20 | 4.14 |
| 28 | 0.10 | 0.03 | 0.20 | 6.13 |
| 29 | 0.12 | 0.03 | 0.19 | 42.78 |

^*^ Position of maximum *F_L_*
