# Supplementary material for: Recent artificial selection in U.S. Jersey cattle impacts autozygosity levels of specific genomic regions
Source: BMC Genomics. 2015 Apr 16;16(1):302. doi: 10.1186/s12864-015-1500-x (PMC4409734; doi:10.1186/s12864-015-1500-x)
Supplement: Additional file 8: — Genomic regions with significant R sb values. This table summarizes all genomic regions with |Rsb| >3. [file 12864_2015_1500_MOESM8_ESM.docx]

**Additional file 8. Genomic regions with significant R_sb_ values.**

| **BTA** | **Range (Mb)** | **Size (Mb)** | **Number of SNP** | **R_sb_^1^** | **Pos^2^ (Mb)** |
| --- | --- | --- | --- | --- | --- |
| 1 | 126.80-127.30 | 0.50 | 11 | 4.48 | 127.17 |
| 2 | 103.32-107.46 | 4.25 | 109 | 7.54 | 106.89 |
|  | 124.22-124.97 | 0.75 | 14 | 7.16 | 124.79 |
|  | 125.03-127.96 | 2.93 | 58 | 7.60 | 125.98 |
| 3 | 4.81-5.83 | 1.02 | 22 | 3.69 | 5.75 |
|  | 37.82-40.05 | 2.23 | 43 | 5.18 | 39.00 |
|  | 40.54-42.76 | 2.22 | 37 | 5.33 | 41.37 |
|  | 96.79-97.67 | 0.88 | 11 | 4.09 | 96.89 |
| 4 | 83.42-84.97 | 1.55 | 31 | 3.98 | 84.11 |
|  | 93.64-94.38 | 0.74 | 20 | 4.04 | 93.98 |
|  | 107.57-108.75 | 1.18 | 20 | 4.27 | 108.23 |
| 5 | 77.00-77.95 | 0.95 | 16 | 4.26 | 77.43 |
| 6 | 84.10-84.50 | 0.40 | 12 | 3.69 | 84.14 |
| 7 | 31.18-35.23 | 4.05 | 78 | 7.81 | 32.61 |
|  | 47.51-48.29 | 0.78 | 13 | 5.70 | 48.21 |
| 13 | 62.86-63.66 | 0.80 | 14 | 4.61 | 63.41 |
| 16 | 59.94-60.47 | 0.53 | 12 | 3.89 | 60.21 |
| 17 | 42.30-43.11 | 0.81 | 14 | 3.51 | 42.79 |
| 18 | 46.41-47.43 | 1.02 | 16 | 3.63 | 47.14 |
| 20 | 3.42-5.74 | 0.52 | 11 | 3.13 | 4.68 |
| 26 | 15.92-18.99 | 1.07 | 19 | 4.91 | 17.76 |
| 1 | 67.63-69.99 | 2.39 | 35 | -5.67 | 68.48 |
|  | 72.35-75.03 | 2.68 | 47 | -6.49 | 73.83 |
| 3 | 84.51-89.00 | 5.49 | 86 | -5.65 | 87.31 |
|  | 90.45-92.96 | 2.51 | 65 | -5.92 | 91.89 |
| 20 | 20.13-23.48 | 3.35 | 59 | -5.01 | 21.27 |
| 29 | 9.26-10.48 | 1.22 | 20 | -3.38 | 10.06 |

^1^ maximum for positive values and minimum for negative values (|R_sb_|>3).

^2^ Position at maximum R_sb._
